# Identifying Abnormal Exertional Breathlessness in COPD: Comparing Modified Medical Research Council and COPD Assessment Test With Cardiopulmonary Exercise Testing

**DOI:** 10.1016/j.chest.2024.10.027

**Published:** 2024-10-28

**Authors:** Magnus Ekström, Hayley Lewthwaite, Pei Zhi Li, Jean Bourbeau, Wan C. Tan, Dennis Jensen, CanCOLD Collaborative Research Group, J. Mark FitzGerald, Don D. Sin, Darcy D. Marciniuk, Denis E. O’Donnell, Paul Hernandez, Kenneth R. Chapman, Brandie Walker, Shawn Aaron, François Maltais, Jonathon Samet, Milo Puhan, Qutayba Hamid, James C. Hogg, Dany Doiron, Palmina Mancino, Pei Zhi Li, Dennis Jensen, Carolyn Baglole, Yvan Fortier, Don Sin, Julia Yang, Jeremy Road, Joe Comeau, Adrian Png, Kyle Johnson, Harvey Coxson, Jonathon Leipsic, Cameron Hague, Miranda Kirby, Mohsen Sadatsafavi, Teresa To, Andrea Gershon, Zhi Song, Andrea Benedetti, Dennis Jensen, Yvan Fortier, Miranda Kirby, Christine Lo, Sarah Cheng, Elena Un, Cynthia Fung, Wen Tiang Wang, Liyun Zheng, Faize Faroon, Olga Radivojevic, Sally Chung, Carl Zou, Palmina Mancino, Jacinthe Baril, Laura Labonte, Kenneth Chapman, Patricia McClean, Nadeen Audisho, Brandie Walker, Curtis Dumonceaux, Lisette Machado, Paul Hernandez, Scott Fulton, Kristen Osterling, Denise Wigerius, Shawn Aaron, Kathy Vandemheen, Gay Pratt, Amanda Bergeron, Denis O’Donnell, Matthew McNeil, Kate Whelan, François Maltais, Cynthia Brouillard, Darcy Marciniuk, Ron Clemens, Janet Baran, Candice Leuschen

**Affiliations:** a Department of Clinical Sciences Lund, Respiratory Medicine, Allergology and Palliative Medicine (M. E.), Faculty of Medicine, Lund University, Lund, Sweden; b Centre of Research Excellence Treatable Traits, College of Health, Medicine and Wellbeing, University of Newcastle, Newcastle, NSW, Australia; c Asthma and Breathing Research Program (H. L.), Hunter Medical Research Institute, Newcastle, NSW, Australia; d Montreal Chest Institute, McGill University Health Center Research Institute, Montréal, QC, Canada; e Translational Research in Respiratory Diseases Program and Respiratory Epidemiology and Clinical Research Unit, McGill University Health Centre Research Institute, Montréal, QC, Canada; f Clinical Exercise and Respiratory Physiology Laboratory, Department of Kinesiology and Physical Education, Faculty of Education, McGill University, Montréal, QC, Canada; g Centre for Heart Lung Innovation, Department of Medicine, University of British Columbia, Vancouver, BC, Canada

**Keywords:** dyspnea, exercise capacity, exercise test, reference values

## Abstract

**Background:**

COPD management is guided by the respiratory symptom burden, assessed using the modified Medical Research Council (mMRC) scale, the COPD Assessment Test (CAT), or both.

**Research Question:**

What are the abilities of mMRC and CAT to detect abnormally high exertional breathlessness on incremental cardiopulmonary cycle exercise testing (CPET) in people with COPD?

**Study Design and Methods:**

Analysis of people aged ≥ 40 years with FEV_1_ to FVC ratio of < 0.70 after bronchodilator administration and ≥ 10 pack-years of smoking from the Canadian Cohort Obstructive Lung Disease study. Abnormal exertional breathlessness was defined as a breathlessness (Borg scale 0-10) intensity rating more than the upper limit of normal at the symptom-limited peak of CPET using normative reference equations.

**Results:**

We included 318 people with COPD (40% female) with a mean (SD) age of 66.5 (9.3) years and FEV_1_ of 79.5% predicted (19.0% predicted); 26% showed abnormally low exercise capacity (peak oxygen uptake less than the lower limit of normal). Abnormally high exertional breathlessness was present in 24%, including 9% and 11% of people with mMRC score of 0 and CAT score of < 10, respectively. An mMRC score of ≥ 2 and CAT score of ≥ 10 was most specific (95%) to detect abnormal exertional breathlessness, but showed low sensitivity of only 12%. Accuracy for all scale cutoffs or combinations was < 65%. Compared with people with true-negatives findings, people with abnormal exertional breathlessness but low mMRC score, low CAT scores (false-negatives findings), or both showed worse self-reported and physiologic outcomes during CPET, were more likely to have physician-diagnosed COPD, but were not more likely to be taking any respiratory medication (37% vs 30%; mean difference, 6.1%; 95% CI, –7.2 to 19.4; P= .36).

**Interpretation:**

In COPD, mMRC and CAT showed low concordance with CPET and failed to identify many people with abnormally high exertional breathlessness.

**Clinical Trial Registry:**

ClinicalTrials.gov; No.: NCT00920348; URL: www.clinicaltrials.gov

**Take-home Points**

**Study Question:** In people with COPD, what is the ability of modified Medical Research Council (mMRC) and COPD Assessment Test (CAT) questionnaires to detect abnormally high exertional breathlessness on incremental cardiopulmonary cycle exercise testing (CPET)?

**Results:** Having an mMRC score of ≥ 2 and CAT score of ≥ 10 was most specific (95%) to detect abnormal exertional breathlessness, but showed low sensitivity of only 12%, and those with abnormal exertional breathlessness on CPET but low mMRC scores, CAT scores, or both (false-negative results) showed worse self-reported and physiologic outcomes during CPET and were more likely to have physician-diagnosed COPD, but were not more likely to be receiving any respiratory medication.

**Interpretation:** The mMRC and CAT assessments showed low concordance with CPET and failed to identify many people with abnormally high exertional breathlessness, supporting the role of standardized exercise testing in the evaluation of people with COPD.

COPD affects about 10% of adults globally and is one of the leading causes of disability and premature death worldwide.^1^^,^^2^ Exertional breathlessness is the cardinal symptom of COPD^1^ and is associated strongly with adverse health outcomes.^3^ Management of COPD is influenced by the individual’s respiratory symptom burden, assessed using the modified Medical Research Council (mMRC) breathlessness scale, the COPD Assessment Test (CAT), or both.^1^^,^^4^ In the most recent Global Initiative for Obstructive Lung Disease (GOLD) guidelines, high respiratory symptom burden (defined as an mMRC rating of ≥ 2 or CAT total score of ≥ 10) mandates intensified treatment such as pulmonary rehabilitation and respiratory medications to improve patient outcomes.^1^

However, people with exertional breathlessness often decrease their level of physical activity to avoid distress from the symptom, which may cause a vicious cycle of physical deconditioning with attendant provocation of more severe and disabling breathlessness at progressively lower levels of physical exertion.^5^ Progressive decreases in physical activity might mask worsening breathlessness when assessed using daily life questionnaires such as the mMRC and CAT.^6^^,^^7^ This potential misclassification could be overcome by assessing exertional breathlessness in relationship to a standardized level of exertion, such as during incremental cycle cardiopulmonary exercise testing (CPET).^6^^,^^7^

CPET is a gold standard method to assess exertional breathlessness in clinical care and research.^8^^,^^9^ Reference equations recently were published for predicting the normal breathlessness intensity response and the upper limit of normal (ULN) at any given power output (measured in Watts), rate of oxygen uptake ($\dot{V}$o_2_), or minute ventilation ($\dot{V}$e) during incremental symptom-limited cycle CPET.^10^ Using these equations, the presence of abnormal exertional breathlessness is defined as a breathlessness intensity (on the Borg 0-10 category-ratio [CR10] scale) > ULN.^10^^,^^11^ Abnormal exertional breathlessness measured at the symptom-limited peak of incremental cycle CPET was shown to have strong and expected associations with a wide range of relevant patient-reported and physiologic outcomes in people with chronic airflow limitation, supporting that the reference equations have strong construct validity for assessing exertional breathlessness.^11^

Emerging evidence suggests that the mMRC scale misclassifies the level of exertional breathlessness in people with COPD^12^^,^^13^ and that undetected (so-called hidden) exertional breathlessness could be unmasked using CPET.^14^^,^^15^ In a study of 92 people referred for incremental cycle exercise testing at a single hospital in Sweden, an mMRC score of ≥ 2 (n = 13 [14%]) identified only 28% of people (9/32) who showed abnormally high breathlessness on the subsequent cycle test.^15^ Thus, the mMRC cut off of ≥ 2 proposed by GOLD^1^ may result in people with abnormally high exertional breathlessness being markedly overlooked, which could contribute to insufficient treatment and worse health outcomes in COPD.

However, to our knowledge, no study has evaluated the ability of the mMRC scale, the CAT, or both to identify abnormal exertional breathlessness assessed using CPET in people with COPD. This investigation would be particularly informative in a population-based sample of people with mostly mild to moderate COPD, who constitute the vast majority of the population with COPD^16^ and in whom respiratory symptoms may remain hidden and undertreated for many years. We aimed to evaluate the ability of the mMRC and CAT questionnaires to identify the presence of abnormal exertional breathlessness assessed using incremental cycle CPET in a population-based sample of people with mostly mild to moderate COPD.^17^

## Study Design and Methods

### Study Design and Population

This was an analysis of the Canadian Cohort Obstructive Lung Disease (CanCOLD) study.^18^ CanCOLD is a prospective study conducted across nine sites in Canada (ClinicalTrials.gov Identifier: NCT00920348), which included noninstitutionalized people aged ≥ 40 years originally identified through random telephone digit dialing.^18^ All participants provided written informed consent before completing study assessments. The research ethics board for each participating institution approved the study protocol.^11^ The current CanCOLD substudy is reported in accordance with the Strengthening the Reporting of Observational Studies in Epidemiology statement.^19^ Part of the current database was used to validate the normative reference equations of breathlessness on CPET.^11^

**Figure 1:**
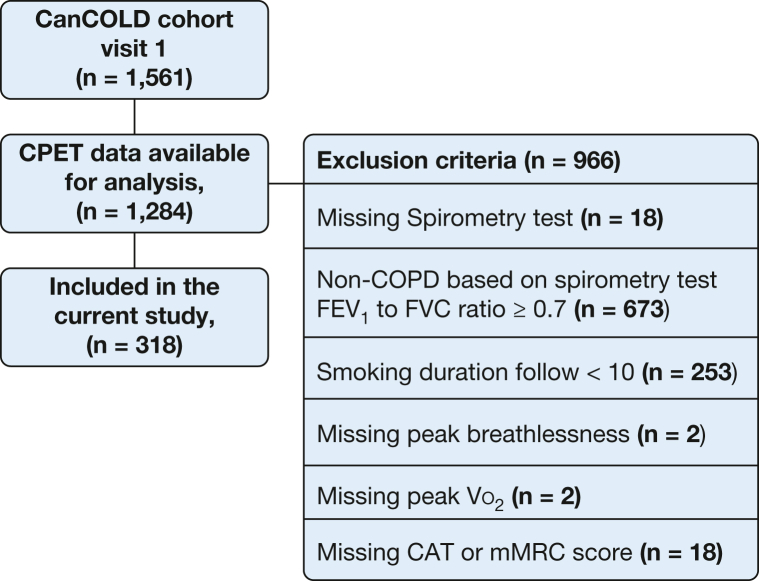
Participant flow diagram. CanCOLD = Canadian Cohort Obstructive Lung Disease; CAT = COPD Assessment Test; CPET = cardiopulmonary exercise testing; mMRC = modified Medical Research Council; $\dot{V}$o_2_ = oxygen uptake.

Inclusion criteria for the present analysis were the presence of COPD, defined in accordance with GOLD as an FEV_1_ to FVC ratio of < 0.70 after bronchodilator administration^1^ and cigarette smoking history of ≥ 10 pack-years, and available data on mMRC, CAT, and CPET at the baseline visit (Fig 1). All eligibility criteria and assessments in the analysis pertain to the CanCOLD baseline visit.

### Assessments and Definitions

Participants self-reported sociodemographics and health information (eg, smoking history and presence of physician-diagnosed health conditions) via structured interview with a trained researcher; health status was assessed using the St George’s Respiratory Questionnaire^20^ and CAT^21^; activity-related breathlessness in daily life using the ordinal mMRC scale of 0 to 4^22^; and physical activity using the Community Healthy Activities Model Program for Seniors questionnaire.^23^ Spirometry (after bronchodilator administration), diffusing capacity of the lungs for carbon monoxide (Dlco), and plethysmographic lung volumes were assessed using automated equipment in accordance with published standards^18^^,^^24^^,^^25^ and were evaluated using Global Lung Function Initiative references.^26^, ^27^, ^28^

### Cardiopulmonary Exercise Testing

CPET was performed in accordance with recognized guidelines on an electronically braked cycle ergometer using a computerized CPET system.^24^ The CPET protocol and assessments were standardized across sites and are detailed in e-Appendix 1.

### Breathlessness Response to CPET

The probability of normality for the observed breathlessness (using the Borg 0-10 category ratio scale breathlessness intensity rating [Borg CR10]) intensity in relationship to $\dot{V}$o_2_ at peak exercise was calculated using published normative reference equations.^10^ The probability of normality corresponds to the predicted probability of observing the Borg CR10 score for healthy people, with lower probability reflecting more abnormal (severe) exertional breathlessness.^10^^,^^11^ The presence of abnormally high exertional breathlessness was defined as a breathlessness intensity rating at peak exercise of more than the ULN.^10^ This methodology has been validated in people with chronic airflow limitation.^11^

### Physiologic Responses to CPET

Physiologic variables evaluated in the current analysis included: exercise capacity as peak W and peak $\dot{V}$o_2_; change in inspiratory capacity (IC) from baseline before exercise to peak exercise expressed in litres (ΔIC) and indexed to peak $\dot{V}$e (ΔIC per peak $\dot{V}$e); nadir of the ventilatory equivalent for carbon dioxide ($\dot{V}$e per rate of exhaled CO_2_), identified as the lowest 30-second average data point observed during CPET; and critical inspiratory constraints as the (1) tidal volume (Vt) to IC ratio and (2) end-inspiratory lung volume to total lung capacity ratio, with both ratios indexed to peak $\dot{V}$e (Vt to IC ratio per peak $\dot{V}$e and end-inspiratory lung volume to total lung capacity per peak $\dot{V}$e). Predicted values for peak power output, peak $\dot{V}$o_2_, and nadir $\dot{V}$e to rate of exhaled CO_2_ were calculated using CanCOLD normative reference equations.^29^

### Statistical Analyses

The mMRC scale was analyzed as ordinal (0-4) scores^22^ and using the cut offs ≥ 1 and ≥ 2.^1^ The CAT total score was analyzed as a continuous variable and using the cut off ≥ 10.^1^ The observed breathlessness intensity (Borg CR10 score) response during the CPET in relationship to peak $\dot{V}$o_2_ % predicted was evaluated using locally estimated scatterplot smoothing plots by each category of mMRC and CAT. Next, the probability of normality for breathlessness was plotted by mMRC and CAT ratings.

Sensitivity, specificity, and accuracy for mMRC and CAT cutoffs to identify abnormally high exertional breathlessness (more than the ULN) on CPET were calculated. The discriminant ability to identify abnormally high exertional breathlessness was evaluated using receiver operating characteristic analysis as the area under the receiver operating characteristic curve. Area under the receiver operating characteristic curve values range from 0.5 (not better than chance) to 1 (perfect), with values of ≤ 0.6 often considered to be poor discrimination.^30^

Differences in characteristics and outcome variables between false-negative and true-negative findings were explored using the Student *t* test and Wilcoxon-Mann-Whitney *U* test for continuous variables with normal and nonnormal distributions, respectively. Categorical variables were analyzed using χ^2^ tests. Statistical significance was defined as a two-sided *P* value of < .05. Statistical analyses were conducted using the SAS version 9.4 software (TS1M5; SAS Institute Inc.).

## Results

### Participants

A total of 318 people with COPD were included in the analysis (Fig 1). The mean age was 66.5 years (SD, 9.3 years; range, 42-89 years), 40% were female, 28% currently smoked tobacco, the mean FEV_1_ was 79.5% predicted (SD, 19.0% predicted) (Table 1), and 26% showed abnormally low exercise capacity (peak $\dot{V}$o_2_ less than the lower limit of normal).

**Table 1:** Characteristics by Presence of Abnormally High Exertional Breathlessness on CPET in 318 People With COPD

Variable	Abnormally High Exertional Breathlessness (> ULN)	Normal Exertional Breathlessness (≤ ULN)
No. of patients	76 (23.9%)	242 (76.1%)
Characteristic		
Age, y		
Mean (SD)	69.1 (8.7)	65.7 (9.4)
Interquartile range	(49.0-85.0)	(42.0-89.0)
Female sex	37 (48.7%)	81 (33.5%)
Body mass, kg	76.0 (15.3)	79.4 (16.5)
BMI, kg/m^2^	27.1 (5.3)	27.3 (4.6)
Cigarette smoking status		
Former	51 (67.1%)	177 (73.1%)
Current	25 (32.9%)	65 (26.9%)
Cigarette smoking duration, pack-years	41.0 (21.4)	37.5 (19.8)
Hypertension	31 (40.8%)	75 (31.0%)
Doctor-diagnosed COPD	35 (46.1%)	75 (31.0%)
Asthma	24 (31.6%)	64 (26.4%)
Any respiratory medications	30 (39.5%)	80 (33.1%)
Self-reported outcomes		
mMRC breathlessness rating		
0	28 (36.8%)	121 (50.0%)
1	35 (46.1%)	106 (43.8%)
≥ 2	13 (17.1%)	15 (6.2%)
CAT total score	10.8 (6.9)	7.6 (6.1)
CAT total ≥ 10	41 (53.9%)	71 (29.3)
SGRQ total score	22.3 (17.2)	15.6 (14.2)
CHAMPS questionnaire, kcal/wk		
Moderate and greater intensity	1,626.0 (1,868.7)	2,274.2 (2,248.1)
All activities	3,416.9 (2,442.0)	4,064.5 (2,910.8)
Lung function at rest		
FEV_1_, % predicted	71.9 (18.2)	81.9 (18.7)
GOLD stage		
1 (FEV_1_ to FVC ratio < 0.7 and FEV_1_ ≥ 80% predicted)	26 (34.2%)	130 (53.7%)
2 (FEV_1_ to FVC ratio < 0.7 and FEV_1_ ≥ 50% and < 80% predicted)	39 (51.3%)	100 (41.3%)
3 (FEV_1_ to FVC ratio < 0.7 and FEV_1_ ≥ 30% and < 50% predicted)	11 (14.5%)	11 (4.5%)
4 (FEV_1_ to FVC < 0.7 and FEV_1_ < 30% predicted)	0 (0.0%)	1 (0.4%)
FVC, % predicted	96.4 (15.4)	103.4 (18.3)
FEV_1_ to FVC ratio, %	57.1 (11.0)	60.9 (7.8)
TLC, % predicted	109.9 (15.8)	109.1 (15.5)
IC, % predicted	93.2 (20.4)	97.2 (21.2)
Dlco, % predicted	78.1 (20.1)	86.4 (22.1)
CPET parameter at peak exercise		
Power output, % predicted	73.1 (19.5)	78.7 (23.6)
Power output < LLN	30 (40.0%)	80 (33.1%)
$\dot{V}$o_2_, % predicted	70.2 (18.7)	82.3 (22.0)
$\dot{V}$o_2_ < LLN	26 (34.2%)	56 (23.1%)
$\dot{V}$e, % predicted	75.5 (19.8)	86.9 (27.9)
Nadir $\dot{V}$e to $\dot{V}$co_2_ ratio	33.9 (6.8)	32.9 (6.9)
Nadir $\dot{V}$e to $\dot{V}$co_2_ ratio > 34	33 (43.4%)	78 (32.2%)
Nadir $\dot{V}$e to $\dot{V}$co_2_ ratio > ULN	23 (30.3%)	57 (23.6%)
Vt to IC ratio per peak $\dot{V}$e	1.62 (0.58)	1.33 (0.50)
EILV to TLC ratio per peak $\dot{V}$e	2.11 (0.76)	1.71 (0.71)
IC difference, L	–0.25 (0.40)	–0.19 (0.41)
IC to $\dot{V}$e ratio difference	–0.006 (0.012)	–0.003 (0.007)
Breathlessness intensity (Borg CR10)	8.0 (5.0-9.0)	4.0 (3.0,6.0)
Leg discomfort (Borg CR10)	8.0 (6.0-9.0)	6.0 (4.0,8.0)
Breathlessness per peak $\dot{V}$e (Borg CR10), /L/min	0.17 (0.06)	0.08 (0.04)
Leg discomfort per peak $\dot{V}$o_2_ (Borg CR10), /L/min	6.68 (2.99)	4.01 (2.32)
Reasons for stopping CPET		
Leg discomfort	22 (29.3%)	121 (51.7%)
Breathlessness	24 (32.0%)	24 (10.3%)
Leg discomfort and breathlessness	20 (26.7%)	32 (13.7%)
Other	9 (12.0%)	57 (24.4%)

Data are presented as No. (%), mean (SD), or median (interquartile range), unless otherwise specified. CAT = COPD Assessment Test; CHAMPS = Community Healthy Activities Model Program for Seniors; CPET = incremental cycle cardiopulmonary exercise testing; CR10 = 0-10 category ratio scale breathlessness intensity rating; Dlco = diffusion capacity of the lungs for carbon monoxide; EILV = end-inspiratory lung volume; GOLD = Global Initiative for Obstructive Lung Disease; IC = inspiratory capacity; LLN = lower limit of normal; mMRC = modified Medical Research Council; SGRQ = St George’s Respiratory Questionnaire; TLC = total lung capacity; ULN = upper limit of normal; $\dot{V}$co_2_ = rate of exhaled CO_2_; $\dot{V}$e = minute ventilation; $\dot{V}$o_2_ = rate of oxygen uptake; Vt = tidal volume.

### Breathlessness Measures

Of 318 participants, 141 participants (44%) and 28 participants (9%) reported an mMRC score of 1 and ≥ 2, respectively, and 112 participants (35%) showed a CAT total score of ≥ 10 (Table 1). Seventy-six participants (24%) had abnormally high exertional breathlessness on CPET. Compared with people with breathlessness on CPET within normal predicted limits, people with abnormally high exertional breathlessness demonstrated more severely impaired lung function (greater airflow limitation, lower Dlco), health status, self-reported physical activity, and exercise capacity (peak $\dot{V}$o_2_), presented with evidence of more severe critical inspiratory constraints at peak exercise, and were more likely to stop exercise because of breathlessness (Table 1).

### Ability of mMRC and CAT to Identify Abnormally High Exertional Breathlessness

**Figure 2:**
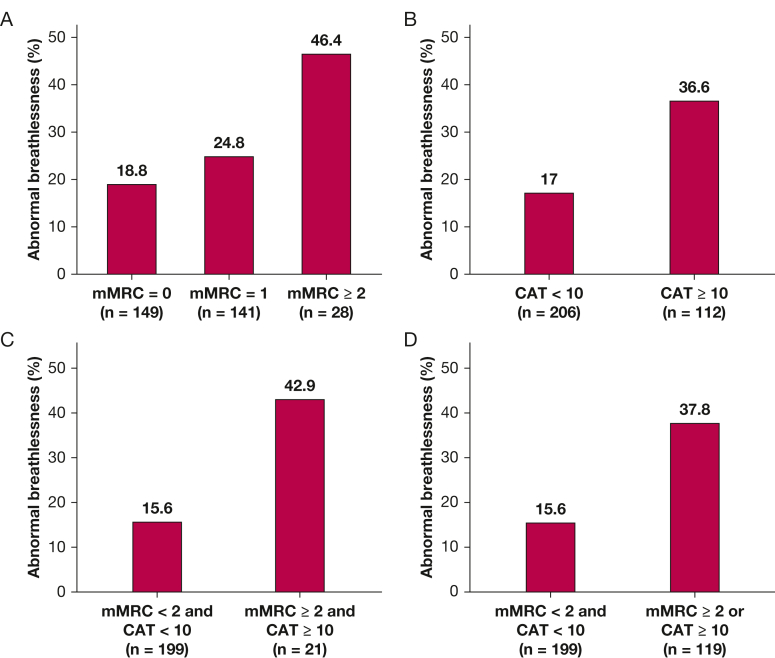
A-D, Bar graphs showing the prevalence of abnormally high exertional breathlessness by mMRC breathlessness rating (A), CAT total score (B), and (C, D) combinations of the scales. CAT = COPD Assessment Test; mMRC = modified Medical Research Council.

Higher mMRC and CAT scores were associated with higher exertional breathlessness intensity (Borg CR10) ratings at the symptom limited peak of CPET (e-Fig 1). However, people classified as having no or low symptom burden (mMRC score < 2 or CAT score < 10) showed highly variable exertional breathlessness intensity responses to CPET, spanning from values well within the normal predicted range to highly abnormal exertional breathlessness (e-Fig 2). Abnormally high exertional breathlessness was present in as many as 18.8% of people with an mMRC score of 0 and in 17% of people with a CAT total score of < 10 (Fig 2). The prevalence of abnormally high exertional breathlessness increased with higher mMRC ratings and CAT total scores (Fig 2): 24.8% for an mMRC rating of 1, 46.4% for an mMRC rating of ≥ 2, and 36.6% for a CAT score of ≥ 10.

**Figure 3:**
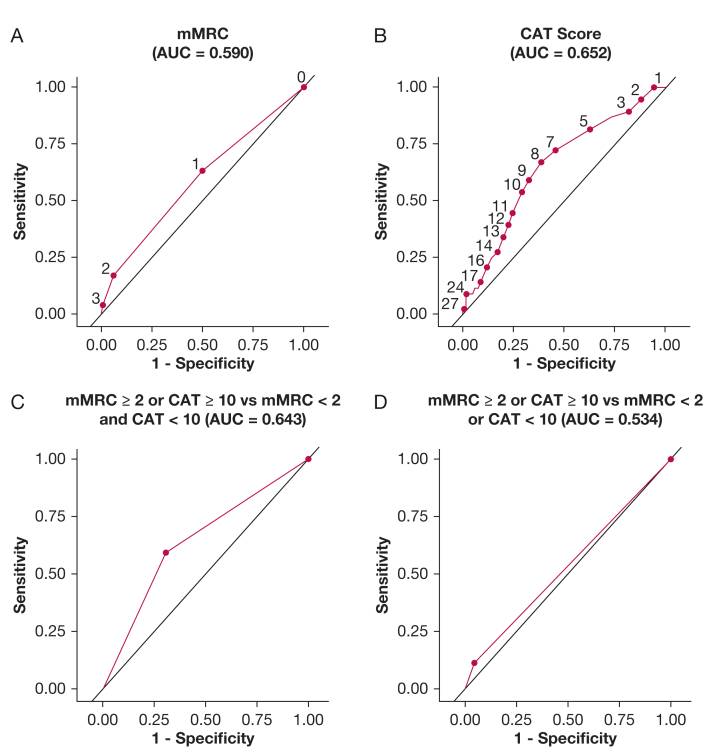
A-D, Line graphs showing detection of abnormally high exertional breathlessness at the symptom-limited peak of incremental cardiopulmonary cycle exercise testing in people with COPD using mMRC breathlessness ratings (A), CAT total scores (B), and combinations of the scales (C, D). The discriminative ability for the variable(s) was assessed as the AUC, where 1.0 is optimal and 0.5 is no better than chance. AUC = area under the receiver operating characteristic curve; CAT = COPD Assessment Test; mMRC = modified Medical Research Council.

The ability of mMRC and CAT scores to detect abnormally high exertional breathlessness is shown in Table 2. An mMRC score of ≥ 2 was the cut off with the highest specificity (94%), but it showed a sensitivity of only 17%, and thus did not identify 83% of people with abnormally high exertional breathlessness on CPET. The highest accuracy (64%) was with an mMRC score of ≥ 2 or a CAT total score of ≥ 10 (Table 2). People who met both GOLD criteria of high respiratory symptom burden (mMRC score ≥ 2 and CAT score ≥ 10) were highly likely to show abnormally high exertional breathlessness on CPET (specificity, 95%), but this combination was insensitive and captured only 12% of people with abnormally high exertional breathlessness (Table 2). The receiver operating characteristic curves in Figure 3 show that all mMRC and CAT cutoffs, both alone and in combination, showed low ability to identify people with COPD who had abnormally high exertional breathlessness on CPET (all areas under the receiver operating characteristic curve, < 0.65).

**Table 2:** Sensitivity, Specificity, and Accuracy of the mMRC Breathlessness Scale and CAT to Identify Abnormally High Exertional Breathlessness (With Symptom-Limited Incremental Cardiopulmonary Cycle Exercise Testing) in 318 People With COPD

Cut Off(s) Used	Abnormally High Exertional Breathlessness (> ULN; n = 76)	Normal Exertional Breathlessness (≤ ULN; n = 242)	Sensitivity (95% CI)	Specificity (95% CI)	Accuracy (95% CI)
mMRC score					
≥ 1	48 (63.2)	121 (50.0)	0.63 (0.52-0.74)	0.50 (0.44-0.56)	0.57 (0.50-0.63)
≥ 2	13 (17.1)	15 (6.2)	0.17 (0.09-0.25)	0.94 (0.91-0.97)	0.55 (0.51-0.60)
CAT total score ≥ 10	41 (53.9)	71 (29.3)	0.54 (0.43-0.65)	0.71 (0.65-0.77)	0.62 (0.56-0.69)
mMRC ≥ 2 or CAT total score ≥ 10	45 (59.2)	74 (30.6)	0.59 (0.48-0.70)	0.69 (0.63-0.75)	0.64 (0.58-0.71)
mMRC ≥ 2 and CAT total score ≥ 10	9 (11.8)	12 (5.0)	0.12 (0.05-0.19)	0.95 (0.92-0.98)	0.53 (0.50-0.57)

Data are presented as No. (%) unless otherwise indicated. Abnormally high exertional breathlessness was defined as a breathlessness intensity (Borg 0-10 category ratio scale breathlessness intensity rating) response more than the ULN at peak exercise during incremental cycle cardiopulmonary exercise testing. The ULN of breathlessness intensity was determined in relationship to peak $\dot{V}$o_2_ using published normative reference equations.^10^ CAT = COPD Assessment Test; mMRC = modified Medical Research Council; ULN = upper limit of normal.

### Classification Concordance and Outcomes

Potential consequences of undetected (or hidden) exertional breathlessness when using mMRC, CAT, or both are explored in Table 3. Compared with patients with true-negative findings (low respiratory symptoms on mMRC and CAT and normal exertional breathlessness on CPET), people with abnormally high exertional breathlessness who were classified as having a low respiratory symptom burden by an mMRC score of < 2 or CAT score of < 10 (false-negative results) showed worse outcomes in terms of airflow limitation, Dlco, self-reported health status, physical activity, and exercise capacity (peak $\dot{V}$o_2_), similar exercise ventilatory inefficiency (nadir $\dot{V}$e per rate of exhaled CO_2_), and more severe critical inspiratory constraints (higher Vt to IC ratio per peak $\dot{V}$e and end-inspiratory lung volume to total lung capacity ratio per peak $\dot{V}$e) in association with higher breathlessness intensity to $\dot{V}$e ratios at peak exercise (Table 3). They also were more likely to have received a diagnosis of COPD from a physician previously, but were not more likely to be taking any respiratory medication (36.5% vs 30.4%; mean difference, 6.1% [95% CI, –7.2 to 19.4]; *P* = .36) (Table 3).

**Table 3:** Outcomes in Relationship to mMRC, CAT, and CPET Ratings of Exertional Breathlessness in 318 People With COPD

Factors	mMRC Score < 2				CAT Total Score < 10				mMRC Score < 2 and CAT Total Score < 10			
	Abnormally High Exertional Breathlessness (> ULN)	Normal Exertional Breathlessness (≤ ULN)	Mean or Proportion Difference (95% CI)	*P* Value	Abnormally High Exertional Breathlessness (> ULN)	Normal Exertional Breathlessness (≤ ULN)	Mean or Proportion Difference (95% CI)	*P* Value	Abnormally High Exertional Breathlessness (> ULN)	Normal Exertional Breathlessness (≤ ULN)	Mean or Proportion Difference (95% CI)	*P* Value
No.	63	227			35	171			31	168		
Age, y			3.1 (0.6-5.7)	.017^a^			2.8 (–0.5 to 6.1)	.097			2.8 (–0.7 to 6.3)	.116
Mean (SD)	68.9 (8.7)	65.7 (9.2)			69.4 (8.6)	66.6 (9.2)			69.4 (8.6)	66.6 (9.2)		
Interquartile range	49.0-84.0	42.0-89.0			49.0-83.0	42.0-89.0			49.0-83.0	42.0-89.0		
Female sex	29 (46.0%)	77 (33.9%)	12.1 (–1.7 to 25.9)	.077	16 (45.7%)	56 (32.7%)	13.0 (–5.0 to 30.9)	.143	15 (48.4%)	56 (33.3%)	15.1 (–3.9 to 34.0)	.108
BMI, kg/m^2^	26.1 (4.4)	27.2 (4.6)	–1.0 (–2.3 to 0.2)	.109	26.6 (4.8)	27.0 (4.5)	–0.4 (–2.0 to 1.3)	.442	26.3 (4.8)	26.9 (4.5)	–0.7 (–2.4 to 1.1)	.293
Smoking status												
Former	43 (68.3)	167 (73.6)	–5.3 (–18.2 to 7.5)	.404	28 (80.0)	134 (78.4)	1.6 (–13.0 to 16.3)	.829	25 (80.6)	132 (78.6)	2.1 (–13.2 to 17.3)	.795
Current	20 (31.7)	60 (26.4)	5.3 (–7.5 to 18.2)	.404	7 (20.0)	37 (21.6)	–1.6 (–16.3 to 13.0)	.829	6 (19.4)	36 (21.4)	–2.1 (–17.3 to 13.2)	.795
Physician-diagnosed COPD	28 (44.4%)	66 (29.1%)	15.4 (1.8-29.0)	.021^a^	11 (31.4%)	37 (21.6%)	9.8 (–6.8 to 26.4)	.212	9 (29.0%)	36 (21.4%)	7.6 (–9.5 to 24.7)	.352
Physician-diagnosed asthma	16 (25.4%)	57 (25.1%)	0.3 (–11.9 to 12.4)	.963	6 (17.1%)	33 (19.3%)	–2.2 (–16.0 to 11.7)	.767	5 (16.1%)	32 (19.0%)	–2.9 (–17.2 to 11.3)	.701
Any respiratory medication	23 (36.5%)	69 (30.4%)	6.1 (–7.2 to 19.4)	.356	10 (28.6%)	39 (22.8%)	5.8 (–10.5 to 22.0)	.466	8 (25.8%)	38 (22.6%)	3.2 (–13.5 to 19.8)	.699
Lung function at rest												
FEV_1_, % predicted	73.0 (18.4)	83.2 (18.1)	–10.3 (–15.4 to –5.2)	< .001^a^	76.4 (17.2)	85.4 (17.5)	–9.0 (–15.4 to –2.6)	.006^a^	77.7 (16.4)	85.7 (17.5)	–7.9 (–14.6 to –1.2)	.02^a^
FVC, % predicted	97.3 (16.1)	104.4 (18.0)	–7.1 (–12.0 to –2.1)	.005^a^	97.9 (16.5)	105.8 (18.3)	–7.9 (–14.5 to –1.3)	.019^a^	98.3 (16.8)	106.1 (18.1)	–7.8 (–14.8 to –0.9)	.026^a^
FEV_1_ to FVC ratio, % predicted	57.3 (10.8)	61.3 (7.2)	–4.0 (–6.3 to –1.7)	.021^a^	60.0 (9.9)	62.1 (6.5)	–2.1 (–4.7 to 0.5)	.794	60.8 (8.6)	62.1 (6.5)	–1.3 (–3.9 to 1.4)	.876
TLC, % predicted	109.4 (15.7)	109.6 (15.4)	–0.2 (–4.8 to 4.4)	.919	109.8 (13.9)	108.7 (15.0)	1.1 (–4.7 to 7.0)	.704	109.2 (14.4)	108.9 (15.0)	0.3 (–6.0 to 6.6)	.924
IC, % predicted	93.0 (21.6)	97.8 (21.1)	–4.8 (–11.1 to 1.4)	.092	97.2 (21.2)	98.1 (21.2)	–0.9 (–9.0 to 7.2)	.603	97.9 (22.2)	98.2 (21.0)	–0.3 (–8.9 to 8.3)	.766
Dlco, % predicted	78.2 (19.4)	87.3 (21.8)	–9.1 (–15.1 to –3.1)	.003^a^	79.3 (18.4)	88.9 (22.3)	–9.5 (–17.5 to –1.6)	.019^a^	79.6 (17.9)	88.9 (22.3)	–9.4 (–17.7 to –1.0)	.029^a^
Self-reported outcomes												
CAT total score	9.6 (5.7)	7.1 (5.7)	2.5 (0.9-4.1)	< 0.001^a^	5.3 (2.7)	4.4 (2.6)	0.9 (–0.0 to 1.9)	.064	5.1 (2.8)	4.4 (2.6)	0.7 (–0.3 to 1.7)	.186
CAT total score ≥ 10	32 (50.8%)	59 (26.0%)	24.8 (11.2-38.4)	< .001^a^	…	…	…	…	…	…	…	…
SGRQ total score	18.3 (14.3)	14.2 (13.1)	4.1 (0.3-7.8)	.018^a^	10.8 (8.7)	9.6 (9.0)	1.2 (–2.1 to 4.5)	.246	9.1 (6.8)	9.4 (8.8)	–0.3 (–3.6 to 3.0)	.636
CHAMPS, kcal/wk												
Moderate and greater intensity	1,806.7 (1,953.4)	2332.2 (2,285.1)	–525.5 (–1147.1 to 96.2)	.075	1,915.0 (1,857.2)	2,362.3 (2,192.0)	–447.3 (–1230.0 to 335.4)	.32	1,990.4 (1,901.8)	2,372.1 (2,206.9)	–381.7 (–1215.6 to 452.2)	.469
All activities	3,620.5 (2,484.6)	4,136.4 (2,927.0)	–515.9 (–1311.2 to 279.4)	.257	3,647.4 (2,517.9)	4,164.0 (2,812.0)	–516.6 (–1528.1 to 494.8)	.272	3,641.2 (2,590.6)	4,169.3 (2,828.2)	–528.1 (–1605.0 to 548.7)	.268
CPET parameters at peak exercise												
Power output, % predicted	75.2 (19.4)	80.1 (23.4)	–4.9 (–11.2 to 1.5)	.193	74.6 (18.7)	83.4 (24.6)	–8.8 (–17.6 to –0.1)	.044^a^	76.8 (18.7)	84.1 (24.4)	–7.3 (–16.5 to 2.0)	.117
$\dot{V}$o_2_, % predicted	71.4 (19.0)	83.4 (21.8)	–12.1 (–18.0 to –6.1)	< .001^a^	72.1 (20.1)	85.7 (23.6)	–13.7 (–22.1 to –5.2)	.002^a^	73.8 (20.4)	86.2 (23.3)	–12.4 (–21.2 to –3.6)	.008^a^
Nadir $\dot{V}$e to $\dot{V}$co_2_ ratio	33.3 (6.5)	32.7 (6.8)	0.6 (–1.3 to 2.4)	.121	33.4 (5.5)	32.3 (6.2)	1.1 (0.0-2.3)	.048^a^	33.2 (5.5)	32.3 (6.2)	0.9 (–1.5 to 3.2)	.0609
Vt to IC ratio per $\dot{V}$e	1.55 (0.48)	1.32 (0.49)	0.23 (0.08-0.37)	< .001^a^	1.61 (0.65)	1.30 (0.50)	0.30 (0.11-0.50)	.003^a^	1.50 (0.41)	1.30 (0.50)	0.19 (0.01-0.37)	.013^a^
EILV to TLC ratio per peak $\dot{V}$e	2.03 (0.72)	1.69 (0.69)	0.35 (0.14-0.55)	< .001^a^	2.06 (0.78)	1.66 (0.72)	0.39 (0.11-0.68)	.002^a^	1.97 (0.64)	1.65 (0.71)	0.32 (0.04-0.61)	.004^a^
IC difference, L	–0.21 (0.38)	–0.19 (0.42)	–0.02 (–0.14 to 0.10)	.753	–0.28 (0.42)	–0.17 (0.40)	–0.11 (–0.27 to 0.04)	.156	–0.24 (0.36)	–0.17 (0.41)	–0.07 (–0.23 to 0.09)	.409
IC to $\dot{V}$e rato difference	–0.004 (0.009)	–0.003 (0.007)	–0.001 (–0.003 to 0.001)	.169	–0.007 (0.015)	–0.003 (0.007)	–0.004 (–0.008 to –0.001)	.044^a^	–0.005 (0.009)	–0.003 (0.007)	–0.002 (–0.005 to 0.001)	.083
Breathlessness per peak $\dot{V}$e (Borg CR10), /L/min	0.16 (0.06)	0.08 (0.04)	0.09 (0.07-0.10)	< .001^a^	0.16 (0.07)	0.07 (0.04)	0.08 (0.07-0.10)	< .001^a^	0.15 (0.05)	0.07 (0.04)	0.08 (0.06-0.09)	< .001^a^
Leg discomfort per peak $\dot{V}$o_2_ (Borg CR10), /L/min	6.36 (2.71)	3.90 (2.21)	2.46 (1.81-3.11)	< .001^a^	6.02 (1.87)	3.82 (2.09)	2.19 (1.44-2.94)	< .001^a^	5.92 (1.58)	3.78 (2.04)	2.15 (1.39-2.91)	< .001^a^

Data are presented as No. (%), mean (SD), unless otherwise indicated. Borg CR10 = Borg 0-10 category ratio scale breathlessness intensity rating; CAT = COPD Assessment Test; CHAMPS = Community Healthy Activities Model Program for Seniors; CPET = cardiopulmonary exercise testing; Dlco = diffusing capacity of the lungs for carbon monoxide; EILV = end-inspiratory lung volume; IC = inspiratory capacity; mMRC = modified Medical Research Council; SGRQ = St George’s Respiratory Questionnaire; TLC = total lung capacity; ULN = upper limit of normal; $\dot{V}$e = minute ventilation; $\dot{V}$o_2_ = oxygen uptake; Vt = tidal volume.

a *P* < .05.

## Discussion

### Main Findings

In this population-based study of relatively asymptomatic people with mostly mild to moderate COPD: (1) mMRC and CAT, alone or in combination, showed low discriminative ability to identify people who had abnormally high exertional breathlessness on standardized cycle CPET; (2) the questionnaires showed especially low sensitivity, meaning that for many people, the exertional breathlessness remained undetected (or hidden); (3) compared with people with breathlessness within normal ranges, people with undetected abnormal exertional breathlessness (false-negative results) experienced worse outcomes in terms of (a) lung function, exercise capacity, self-reported health status, and physical inactivity, (b) more severe critical inspiratory constraints at peak exercise, and (c) being not more likely to have received any respiratory medication, despite being more likely to have received a prior physician diagnosis of COPD.

### Strengths and Limitations

A strength of this study is the use of a well-characterized, population-based cohort of people with COPD. The population sample decreased the risk of selection bias compared with clinical cohorts, captured a target population for early detection of COPD and exertional breathlessness, and increased external validity of the findings because people with mild to moderate airflow limitation constitute about 95% of all people with COPD.^16^ The limitation that few people had severe airflow limitation should be addressed in future studies. Further, the present study included only people with COPD older than 40 years who reported a smoking history of ≥ 10 pack-years. Further research is needed to understand better symptom profiles and exertional breathlessness in people who demonstrate COPD early in life, as a result of non-smoking-related factors, or both.^31^ Nevertheless, CanCOLD provides unique data on symptoms in daily life self-rated using validated questionnaires,^22^^,^^32^ detailed physiologic measurements, and standardized CPET.^18^ Breathlessness abnormality and physiologic responses were categorized using normative reference equations developed in CanCOLD.^10^^,^^29^

### Clinical Implications

This study showed that in mild to moderate COPD, people with an mMRC score of ≥ 2 are likely to be symptomatic (high specificity), but that mMRC and CAT, used alone or in combination, have low sensitivity and fail to identify most people with high exertional breathlessness. Many patients who self-report having no or mild symptoms on the questionnaires will demonstrate markedly abnormally increased exertional breathlessness on a standardized CPET. An inherent limitation of the mMRC and CAT questionnaires is that they invite participants to recall the intensity or impact of any one or combination of symptoms in their daily life, without accounting for the level of exertion (stimulus) that provoked the symptoms. People are likely to adapt their level of activity based on their symptoms, which can mask the true intensity of exertional breathlessness and can impact on daily life physical activity. This was indicated in the present study when people with an mMRC score of 0 or 1, but abnormally high exertional breathlessness on CPET (ie, people with so-called hidden exertional breathlessness), self-reported significantly lower physical activity compared with people with exertional breathlessness within normal predicted limits. For the clinician, the present findings suggest that a self-rated mMRC score of ≥ 2 is specific and likely to reflects the presence of abnormally high exertional breathlessness, possibly mandating intensified treatment in accordance with the GOLD guidelines.^1^

The high percentage of undetected abnormal exertional breathlessness suggests that standardized exercise testing may play an important (and underused) role in the clinical evaluation of people with COPD. A lower resource-intensive option compared with CPET is the 6-min walk test, which is widely accessible and inexpensive.^33^ Indeed, normative reference equations to evaluate the presence and level of abnormal exertional breathlessness in response to a 6-min walk test were published recently.^34^ Other field tests to assess exertional breathlessness at a standardized level of exertion are the 3-min constant-rate stair stepping test and the shuttle walking test.^35^, ^36^, ^37^ These tests were developed mainly to measure change in exertional breathlessness in people with COPD and need further validation for categorizing level of symptom severity.^8^ Because of higher costs and more limited availability compared with field tests, we suggest that incremental CPET may be reserved for people with unexplained problematic symptoms in whom the underlying physiologic mechanisms are not clear and for whom the CPET results are more likely to influence clinical practice.^8^^,^^38^

Improved assessment of exertional breathlessness is important because we found that people with undetected abnormal exertional breathlessness (compared with those with exertional breathlessness within normal limits) showed worse airflow limitation, lower exercise capacity, and higher breathlessness intensity to $\dot{V}$e ratios in association with more severe critical inspiratory constraints at peak exercise, all of which are treatable traits that are likely to be responsive to inhaled respiratory medications.^1^^,^^39^^,^^40^ Undertreatment and the need for improved assessment was highlighted further by the fact that among of these people with undetected exertional breathlessness—despite having worse health status and lung function and being more likely to have been received a diagnosis of COPD—only 35% were receiving any respiratory medication. This discrepancy might reflect in part that they were classified by the mMRC and CAT questionnaires as having a low respiratory symptom burden. Therapeutic trials targeting people with COPD and so-called hidden exertional breathlessness are needed to test this hypothesis.

### Research Implications

This study supports that an mMRC rating of ≥ 2 is the most accurate cut off to identify the presence of abnormally high exertional breathlessness in epidemiologic studies. However, the burden of exertional breathlessness assessed using the mMRC and CAT questionnaires might be misclassified and underestimated. In the present study, of people with mostly mild to moderate COPD originally recruited from the general population, about 20% with a mMRC score of 0 or 1 or a CAT score of < 10 had abnormally high exertional breathlessness on CPET.

Next research steps include further validation of the present findings in clinical populations with more severe illness, as well as in health conditions other than COPD. The present findings are likely to have important implications in other cardiopulmonary conditions marked by progressive exertional breathlessness, such as in interstitial lung disease, pulmonary hypertension, and heart failure, for which current management is based on symptom assessment using the New York Heart Association scale,^41^ a questionnaire that is very similar to the mMRC breathlessness scale. An important next step will be to validate a stepwise approach to evaluate the presence and severity of exertional breathlessness involving clinical factors and standardized exercise testing (CPET, 6-min walk test) to optimize evaluation and treatment of symptoms in people with chronic cardiopulmonary disease.

## Interpretation

In people with mostly mild to moderate COPD, the mMRC and CAT questionnaires were shown to have low concordance with CPET to identify abnormally high exertional breathlessness. A mMRC score of ≥ 2 had high specificity and may be useful for screening, but identified only 17% of the patients who showed abnormally high breathlessness on CPET. Standardized exercise testing may be useful to improve symptom assessment and management in COPD.

## Funding/Support

The CanCOLD study (ClinicalTrials.gov Identifier: NCT00920348) has received support from the Canadian Respiratory Research Network, the Canadian Institutes of Health Research [CIHR/Rx&D Collaborative Research Program Operating Grant 93326], the Respiratory Health Research Network of the Fonds de la Recherche en Santé du Québec, the Foundation of the McGill University Health Centre, and industry partners, including: AstraZeneca Canada, Ltd., Boehringer Ingelheim Canada, Ltd., GlaxoSmithKline (GSK) Canada, Ltd., Novartis, Almirall, Merck, Nycomed, Pfizer Canada, Ltd., and Theratechnologies. M. E. was supported by an unrestricted grant from the Swedish Research Council [Dnr: 2019-02081]. D. J. holds a Canada research chair, tier II, in clinical exercise and respiratory physiology from the Canadian Institutes of Health Research.

## Financial/Nonfinancial Disclosures

The authors have reported to *CHEST* the following: J. B. and W. C. T. report receiving institutional funding for the CanCOLD study from Astra Zeneca Canada, Ltd., Boehringer-Ingelheim Canada, Ltd., GlaxoSmithKline Canada, Ltd., Merck, Novartis Pharma Canada, Inc., as well as Nycomed Canada, Inc. (W. C. T.), Pfizer Canada, Ltd. (W. C. T.), Trudell (J. B.), and Grifolds (J. B.). Unrelated to this work, M. E. has received a research grant from ResMed and personal fees from AstraZeneca, Boehringer Ingelheim, Novartis, and Roche. None declared (H. L., P. Z. L., D. J.).

## Supplementary Data

e-Online Data

e-Online Data

e-Online Data
