# Supplementary material for: Identifying Abnormal Exertional Breathlessness in COPD: Comparing Modified Medical Research Council and COPD Assessment Test With Cardiopulmonary Exercise Testing
Source: Chest. 2024 Oct 28;167(3):697–711. doi: 10.1016/j.chest.2024.10.027 (PMC11882773; doi:10.1016/j.chest.2024.10.027)
Supplement: e-Online Data [file mmc1.docx]

**e-Figure 1.** Breathlessness intensity (Borg CR10) ratings during the symptom limited incremental cardiopulmonary cycle exercise test in relation to V’O_2_%pred_max_, by a) modified Medical Research Council (mMRC) breathlessness ratings; and b) COPD Assessment Test total scores.
