# Supplementary material for: Identifying Abnormal Exertional Breathlessness in COPD: Comparing Modified Medical Research Council and COPD Assessment Test With Cardiopulmonary Exercise Testing
Source: Chest. 2024 Oct 28;167(3):697–711. doi: 10.1016/j.chest.2024.10.027 (PMC11882773; doi:10.1016/j.chest.2024.10.027)
Supplement: e-Online Data [file mmc2.docx]

**e-Figure 2.** Probability of breathlessness normality at peak exercise during incremental cycle cardiopulmonary exercise testing (CPET), by self-rated modified Medical Research Council (mMRC) breathlessness score and COPD Assessment Test (CAT) total score. The probability of normality was calculated using published normative reference equations in relation to the rate of oxygen uptake in percent of the predicted maximum for the individual (V’O_2_%pred_max_) at peak exercise.[^1^](#_ENREF_1)^,^[^2^](#_ENREF_2) The probability of normality corresponds to the probability of the breathlessness intensity rating among healthy references, with a lower probability interpreted as more abnormal (severe) breathlessness. The distributions show that people with higher mMRC and CAT scores were more likely to have more abnormal breathlessness during CPET. However, people with low scores on mMRC or CAT had highly variable breathlessness intensity responses during CPET, spanning from values well within the normal predicted range to highly abnormal exertional breathlessness

**References**

1 Ekström M, Li PZ, Lewthwaite H, Bourbeau J, Tan WC, Schiöler L, Brotto A, Stickland MK, Jensen D. Normative reference equations for breathlessness intensity during incremental cardiopulmonary cycle exercise testing in people aged 40 years and older. Annals of the American Thoracic Society 2023. *In press.*

2 Ekström M, Li PZ, Lewthwaite H, Bourbeau J, Tan WC, Jensen D, On behalf of the CanCOLD Collaborative Research Group. Abnormal exertional breathlessness on cardiopulmonary cycle exercise testing in relation to self-reported and physiological responses in chronic airflow limitation. Chest 2024. *In press.*
