# Supplementary material for: Identifying Abnormal Exertional Breathlessness in COPD: Comparing Modified Medical Research Council and COPD Assessment Test With Cardiopulmonary Exercise Testing
Source: Chest. 2024 Oct 28;167(3):697–711. doi: 10.1016/j.chest.2024.10.027 (PMC11882773; doi:10.1016/j.chest.2024.10.027)
Supplement: e-Online Data [file mmc3.docx]

**ONLINE SUPPLEMENT**

**Identifying abnormal exertional breathlessness in COPD: comparing mMRC and CAT with CPET**

**e-Appendix 1. CPET protocol and assessments**

The incremental cardiopulmonary cycle exercise test (CPET) protocol and assessments were standardized across sites and included a steady state pre-exercise baseline period of three to 10 minutes, followed by one minute of unloaded pedalling, and then 10 W/min increases in power output (starting at 10 W) until symptom limitation.

Gas exchange and breathing pattern parameters were collected breath-by-breath with participants breathing through a mouthpiece and flow transducer while wearing a nose clip. Heart rate (HR) and rhythm were assessed continuously by 12-lead ECG, and peripheral oxyhemoglobin saturation (SpO_2_) was monitored by finger pulse oximetry. At rest, every two minutes during exercise, and at peak exercise, participants performed maximal voluntary inspiratory capacity (IC) maneuvers [^1^](#_ENREF_1), and rated the intensity of their perceived breathlessness and leg discomfort using the Borg CR10 scale.[^2^](#_ENREF_2) Prior to CPET, breathlessness was defined for each participant as “breathing discomfort” and leg discomfort as “the level of leg discomfort experienced during pedalling”; and participants were familiarized with Borg’s CR10 scale such that “0” represented “no breathing (leg) discomfort” and “10” represented “the most severe breathing (leg) discomfort that you have ever experienced or can imagine experiencing”. Peak W was taken as the highest power output a participant was able to sustain for ≥30-s, whereas peak V’O_2_ and V’_E_ were taken as the average of the breath-by-breath data over the last 30-s of loaded pedalling.

**References**

1 Guenette JA, Chin RC, Cory JM, et al. Inspiratory Capacity during Exercise: Measurement, Analysis, and Interpretation. Pulm Med 2013; 2013:956081

2 Burdon JG, Juniper EF, Killian KJ, et al. The perception of breathlessness in asthma. Am Rev Respir Dis 1982; 126:825-828
